# The prevalence and under-diagnosis of vertebral fractures on chest radiograph

**DOI:** 10.1186/s12891-018-2171-y

**Published:** 2018-07-18

**Authors:** Yizhong Li, Lisheng Yan, Siqing Cai, Peiwen Wang, Huafeng Zhuang, Haiming Yu

**Affiliations:** 1 Department of Orthopedics, the Second Affiliated Hospital of Fujian Medical University, Quanzhou, 362000 Fujian China; 2 The Department of Radiology, The Second Affiliated Hospital of Fujian Medical University, Zhongshan North road 34, Quanzhou, 362000 Fujian China

**Keywords:** Vertebral fracture, Prevalence, Missed diagnosis, Chest radiograph

## Abstract

**Background:**

Vertebral fracture is the most common fragility fracture but it remains frequently unrecognized and is underdiagnosed worldwide. In this retrospective study, we examined the prevalence of moderate and severe vertebral fractures on chest radiographs of hospitalized female patients aged 50 years and older and determined missed diagnosis of vertebral fractures in the original radiology reports.

**Methods:**

3216 female patients 50 years of age and older were enrolled in our study. The patients’ medical records including their original radiology reports and lateral chest radiographs were retrospectively reviewed by the study radiologists who had training certificates from the International Society for Clinical Densitometry (ISCD). Vertebral fractures between thoracic spine T_4_ and lumbar spine L_1_ were identified and classified using Genant’s semi-quantitative scale. The definition of vertebral fractures used in this study was Genant grade 2 or higher.

**Results:**

The study radiologists identified 295(9.2%) patients with grade 2 or 3 fractured vertebrae, total 444 vertebrae on 3216 chest radiographs. The prevalence of vertebral fracture was 2.4% in women aged 50-59 yrs., 8.9% in women aged 60–69 yrs., and 21.9% in women aged≥70 yrs. There were 213 patients with a single vertebral fracture, 49 patients with two vertebral fractures and 33 patients with ≥ three vertebral fractures. Fractured vertebrae were identified with greater frequency in thoracic spine T_11,12_ and lumbar spine L_1_. According to our statistics, 66.8% of patients with vertebral fractures found in this study were undiagnosed in the original radiology reports.

**Conclusions:**

Vertebral fracture is common on chest radiographs but it is often ignored by radiologists. Genant’s semiquantitative assessment is a simple and effective method for detecting vertebral fracture. Because osteoporotic vertebral fracture increases the risk of new fractures, radiologists have an important role in accurately diagnosing vertebral fractures.

## Background

Vertebral fracture is the most common fragility fracture and is a hallmark of osteoporosis. These fractures frequently occur in the elderly. The incidence of new vertebral fracture in women and men aged 50 years and over was 10.7/1000 person years and 5.7/1000 person years respectively in Europe [1]. Clinical vertebral fractures were estimated to be 1.4 million around the world in 2000 [2]. 520,000 new vertebral fractures were sustained in the 27 countries of the European Union (EU27) in 2010 [3].The incidence of vertebral fracture markedly increases with age in both female and male. The prevalence of vertebral fracture increased from 3% in women below 60 years of age to 20% in women aged over 70 yrs. and from 7.5 to 20% in men over the same age range [4]. The incidence rate of new vertebral fracture in Chinese men and women aged 50 and above was 194/100,000 person-years and 508/100,000 person-years respectively in Hong Kong [5]. In Beijing of China, the prevalence of vertebral fracture increased from 15% in women aged over 50 years to 36.6% in women aged over 80 yrs. [6]. Osteoporotic vertebral fracture is a marker of reduced bone strength and the presence of existing vertebral fracture increases the risk of new fractures and death. The accurate diagnosis of vertebral fractures is important for the treatment of osteoporosis and prevention of new fractures. Because many vertebral fractures are asymptomatic or cause mild pain, the majority of vertebral fractures are not diagnosed worldwide [7]. Vertebral fractures need radiological confirmation but are often undiagnosed by radiologists, with a misdiagnosis rate up to 50% [8]. In this study, we examined the prevalence of moderate and severe vertebral fractures on the chest radiographs of hospitalized patients and determined missed diagnosis of vertebral fractures in the original radiology reports.

## Methods

### Study sample

This retrospective research enrolled 3216 female patients 50 years of age and older who were admitted to the Second Affiliated Hospital of Fujian Medical University in China from January 2014 to December 2015 and had the lateral chest radiographs taken during hospitalization. A total of 3600 patients’ medical records including original radiology reports and lateral chest radiographs were retrospectively reviewed. 384 patients were excluded by history and/or by observations on radiographs because of vertebral fracture due to high-energy trauma, post-traumatic deformity, admission with the diagnosis of osteoporosis, metastatic tumors, tuberculosis, Scheuermann’s disease, congenital spine deformity, deformity due to degenerative scoliosis or an unclear image of lateral thoracic spine. 3216 patients in this study were between 50 to 95 years of age, with an average age of 62.3 ± 9.7 yrs. Overall, 1310 patients were aged 50–59, 1180 patients were aged 60–69 and 726 patients were aged ≥70 yrs. Approval for this research was given by the ethics committee of the Second Affiliated Hospital of Fujian Medical University.

### Identification of prevalent vertebral fracture

The digital radiographs of lateral chest were reviewed and reported respectively by 2 study radiologists who were Certified Clinical Densitometrist conferred by the International Society for Clinical Densitometry (ISCD). A consensus was reached between the radiologists for any difference of interpretation. Vertebral fractures between thoracic spine T_4_ and lumbar spine L_1_ were identified by visual inspection and classified using Genant’s semi-quantitative scale [9]. Vertebral fractures identified in this study were defined as Genant grade 2 or higher. A loss of vertebral height of 25–40% was defined as grade 2(moderate) and a loss greater than 40% was defined as grade 3(severe). Vertebral height losses of 20–25%(grade 1, mild) were not calculated as vertebral fractures in our study, because mild vertebral deformities included some variants or remodeling deformities such as physiological wedging in the mid thoracic region and short vertebral height due to increasing age and degenerative change [10]. Including mild fractures carries greater sensitivity and lower specificity.

### Statistical analysis

The data was presented as mean, standard deviation, and percentage. All statistical analyses were performed using SPSS statistical software (SPSS, version19.0; SPSS Inc., Chicago, Illinois). All groups parameters were compared with a chi-square test of R^χ^C table data. *P* < 0.05 was considered as statistically significant.

## Results

**Fig. 1:**
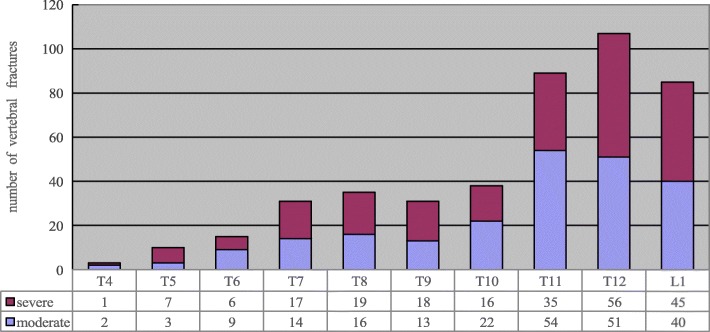
Distribution of fractures by vertebral level between the thoracic spine T_4_ and lumbar spine L_1_ by Genant’s semi-quantitative scale

The study radiologists identified 295(9.2%) patients with 444 grade 2 or 3 fractured vertebrae on 3216 chest radiographs. The prevalence of vertebral fracture was 2.4% in women aged 50-59 yrs.,8.9% in women aged 60–69 yrs., and 21.9% in women aged≥70 yrs. (Table 1).157 patients had ≥ one grade 2 vertebral fracture with a total of 200 fractured vertebrae, while 138 patients had ≥ one grade 3 vertebral fracture with a total of 244 fractured vertebrae. There were 213 patients with single vertebral fracture, 49 patients with two vertebral fractures and 33 patients with ≥ three vertebral fractures (Table 2). Fractured vertebrae occurred with greater frequency in thoracic spine T_11,12_ and lumbar spine L_1_(Fig. 1).

**Table 1:** Impact of age on the prevalence of vertebral fracture in the female aged ≥50 yrs.

	50–59 yrs	60-69 yrs	*≥*70 yrs
Patients	1310	1180	726
Cases of vertebral fracture			
n, (%)	31(2.37%)#△	105(8.90%)*△	159(21.90%)*#

*comparison with group of 50–59 yrs., *p* < 0.05

#comparison with group of 60–69 yrs., *p* < 0.05

△comparison with group of ≥70 yrs., *p* < 0.05

**Table 2:** Characteristics of vertebral fractures identified on chest radiographs

	Number of patients (*n*)	Moderate fractured vertebrae (*n*)	Severe fractured vertebrae (*n*)
One vertebral fracture	213	132	81
Two vertebral fractures	49	42	56
≥ three vertebral fractures	33	50	83
Total	295	224	220

Among the 295 patients with vertebral fractures identified in this study, only 98 had vertebral fractures documented in their original radiology reports. These vertebral fractures were described by imprecise terms such as “biconcavity” or “wedge deformity” and their severity was no annotated. Thus, the missed diagnosis of vertebral fracture was as high as 66.8%.

## Discussion

The prevalence of vertebral fracture was 9.2% in our study and increased with age in female aged ≥50 yrs. Vertebral fractures occurred primarily in the thoracolumbar junction. Overall, 66.8% of patients with vertebral fractures found in this study were undiagnosed in their original radiology reports.

The 9.2% fracture prevalence in our study was lower than the 11–14% prevalence reported in other studies of chest radiographs [8, 11]. The reasons for lower fracture prevalence in our study likely reflect our stricter criteria of only including moderate or severe vertebral fractures between thoracic spine T_4_ and lumbar spine L_1_ and the the younger age of our patient cohort. In the study reported by Gehlbach SH, the mean age of the study patients was 75.9 yrs. and lumbar spine L_2_ was included [8]. In the study reported by Lansdown D, the mean age of the study patients was 74.5 yrs. in Caucasian and 74.9 yrs. in African American women, and the mild vertebral fractures were also included [11].

### Under-diagnosis of vertebral fracture

Under-diagnosis of vertebral fracture is a worldwide problem. In a multinational study of assessing the accuracy of radiographic diagnosis of vertebral fractures, the false-negative rates were 34% globally (from 27 to 45%), despite good image quality, the same semiquantitative technique and the quality assurance manual for fracture assessment [7]. A retrospective study of 934 hospitalized women aged 60 years and older identified 132 patients with moderate or severe vertebral fractures in chest radiographs and showed that only half of the contemporaneous radiology reports identified these fractures [8]. In a study of the prevalence of vertebral fractures on routine chest radiographs of 1011 women over 60 years of age, only 18% of patients with moderate or severe vertebral fractures found in the study had vertebral fractures mentioned in the original radiology reports [11]. The pervasive radiologic under-diagnosis of vertebral fracture occurs similarly in China. A retrospective study of 1638 hospitalized patients aged 50 years and older showed that 64% of patients with vertebral fractures found on routine chest radiographs were undiagnosed in the original radiology reports [12]. These results were in line with our study, where 66.8% of patients with vertebral fractures were undiagnosed in the original radiology reports. Moderate or severe vertebral fracture means the obvious deformity of vertebral body. Why are so many vertebral fractures missed by radiologists? Possible explanations include: 1) radiologists often focus on more acute, significant and urgent findings related to the heart and lungs, 2) radiologists lack sufficient knowledge of osteoporosis and sufficient training for fracture recognition, 3) radiologists diagnose vertebral fractures according to the characteristic findings of traumatic fracture and do not recognize deformed vertebrae as manifestations of osteoporotic fracture, and 4) radiologists ignore the presence of vertebral fractures.

### Improvement of the radiological diagnosis of vertebral fracture

How can we improve the radiological diagnosis of vertebral fracture? Because many fragility vertebral fractures are present on radiographs obtained for other reasons and are unsuspected clinically, radiologists must use a standardized approach to the diagnosis of vertebral fracture, such as vertebral fracture assessment. Genant’s semiquantitative assessment and quantitative assessments are two major approaches to the identification of vertebral fracture. Both approaches have moderately sensitivity and specificity for identifying vertebral fracture and have high reproducibility. Previous work has shown that there are no significant differences in detecting vertebral fractures between the two methods [13]. Purely quantitative morphometric assessment relies entirely upon placing six points in each vertebral body to calculate the anterior, middle, and posterior heights and their ratios. One area of concern with this approach is that thoracic vertebrae are usually wedge-shaped and might be falsely diagnosed as mild fracture by morphometric assessment, especially in the presence of kyphosis due to osteoarthritis for example. Thus, quantitative morphometric assessment is not recommended in routine clinical settings. Genant’s semiquantitative assessment is a basis for a standardized interpretation of vertebral fracture severity and is simple for radiologists and orthopedic surgeons to learn and apply in clinical practice. Genant’s semiquantitative assessment of vertebral fractures can be performed on lateral radiographs of the thoracolumbar spine or lateral spine images obtained with dual-energy X-ray absorptiometry (DXA), and has been recommended for identification of vertebral fractures by ISCD [14]. Chest radiographs are frequently executed for hospitalized patients and the image of thoracic vertebra T_4_ to lumbar vertebra L_1_ is clear in lateral chest radiographs. The high under-diagnosis rate in original radiographic reports suggests that many general radiologists do not follow the ISCD recommendation. It is pressing that the ISCD recommendation for vertebral fracture assessment and update advances in osteoporosis need to be known by radiologists, orthopedic surgeons and physicians who are not specialized in osteoporosis. The increased rate of vertebral fractures identified by study radiologists in this study suggests that improved education and training regarding osteoporotic fracture could augment the accurate radiological diagnosis of vertebral fractures.

### Effect of assessing vertebral fractures

Osteoporosis is diagnosed by low bone mass and by fragility fracture including vertebral fracture and hip fracture. Diagnosis of severe osteoporosis requires a T-score of bone mineral density(BMD) ≤ − 2.5 plus at least one fragility fracture. Many vertebral fractures are already present in patients with a T-score of BMD > − 2.5. Several studies have confirmed that the combination of BMD and vertebral fracture assessment significantly increases diagnostic rate of osteoporosis from 9.8–13.4 and 14.7% for severe osteoporosis [15–18]. The terms “vertebral fracture” and “severe osteoporosis” will likely attract the attention of both orthopedic surgeons and patients. The presence of a vertebral fracture increases the relative risk of future vertebral fracture by 4.4 fold and the risk of hip fracture by two fold [19]. Moreover, the greater the number and severity of prevalent vertebral fractures, the greater the risk of future vertebral fracture [20, 21]. Prevention of new fracture is paramount for patients with existing vertebral fracture. Fragility vertebral fracture is one of indications for initiation of anti-osteoporosis drug therapy. Several drug therapies have proved to reduce the risk of vertebral fractures by 30–65% and future fracture risk [22]. Early identification of vertebral fracture can improve the diagnosis and treatment of osteoporosis.

There are some limitations in our study. We only examined chest radiographs and included moderate and severe vertebral fractures. Therefore, the prevalence of vertebral fracture was likely lower than real prevalence. We also did not delineate the relationship between vertebral fracture and BMD in our patients. With these limitations in mind, this study identified many vertebral fractures that were undiagnosed in original radiology reports and underscores the importance of utilizing vertebral fracture assessment in general practice.

## Conclusions

Vertebral fracture is common on chest radiographs and is frequently unrecognized. Genant’s semiquantitative assessment is an effective method for detecting vertebral fracture. Radiologists have an important role in the accurate diagnosis of vertebral fracture.

## Abbreviations

BMD: Bone mineral density
DXA: Dual-energy X-ray absorptiometry
ISCD: International Society for Clinical Densitometry
